# *Drosophila* Populations Reared Under Tropical Semi-natural Conditions Evolve Season-dependent Differences in Timing of Eclosion

**DOI:** 10.3389/fphys.2022.954731

**Published:** 2022-07-15

**Authors:** Chitrang Dani, Vasu Sheeba

**Affiliations:** Chronobiology and Behavioural Neurogenetics Laboratory, Neuroscience Unit, Jawaharlal Nehru Centre for Advanced Scientific Research, Bengaluru, India

**Keywords:** evolution, circadian, temperature, humidity, light, drosophila, natural, eclosion

## Abstract

Circadian clocks are considered an evolutionary adaptation to environmental cycles, helping organisms to adapt to daily and seasonal changes. However, most studies on the evolution of circadian rhythms have been carried out in controlled laboratory conditions; hence evolution of circadian clocks and rhythms in organisms reared under the influence of naturally varying time cues is not well understood. To address this, we reared large outbred fly populations in an outdoor enclosure on our institutional grounds in Bengaluru, southern India for about 150 generations, at the same time maintaining their ancestral control populations under standard laboratory conditions. Studying their rhythms in eclosion, a vital behavior for *Drosophila*, in the laboratory and semi-natural environments revealed that flies reared under semi-natural conditions differed in the timing of eclosion under semi-natural conditions in a season-dependent manner from their laboratory-reared counterparts. These differences were manifested under harsh semi-natural environments but not under mild ones or in standard laboratory conditions. Further analysis revealed that this phenotype might be responsive to seasonal changes in temperature cycles which was confirmed in the laboratory with simulated light and temperature cycles that approximated semi-natural conditions. Our results highlight key intricacies on the relative impact of intensity and timing of environmental cues for predicting the timing of *Drosophila* eclosion under tropical naturalistic conditions. Overall, our research uncovers previously unexplored aspects of adaptive circadian timekeeping in complex natural conditions, offering valuable insight into the evolution of clocks.

## Introduction

Circadian clocks are internal timekeepers, with intrinsic periodicities of ∼24 h and synchronize to certain daily cyclic changes in the environment (zeitgebers) through the process of entrainment. Circadian clocks are found to drive rhythms in behavior, physiology and metabolism in several species and are thought to have evolved multiple times independently during the course of evolution (Dunlap et al., 2004). These internal timers have also been demonstrated to persist and function despite long-term rearing under aperiodic conditions (Sheeba et al., 2002; Imafuku and Haramura, 2011). They are characterized by properties such as free-running period (innate periodicity exhibited by individuals under constant conditions) and phase-angle of entrainment (timing of behavioral or physiological events with respect to the external cycles). These circadian clock properties exhibit variation across species (Pittendrigh and Daan, 1976) and may even represent adaptations to local environments within a species (Daan, 1981). The range of entrainment, phase-angle (ψ) and entrained amplitudes have been shown to vary systematically not only with respect to the zeitgeber but also with intrinsic clock properties (Aschoff, 1960; Winfree, 2001; Schmal et al., 2020).

Eclosion is a critical event for the fruit fly *Drosophila melanogaster*, and as it occurs only once in the lifetime of an individual insect, rhythmic eclosion requires synchronized emergence of several adults from pupae. Thus, the eclosion rhythm is inherently a population-level rhythm, its waveform reflecting average population behavior and inter-individual variation in the population. The eclosion rhythm was one of the earliest rhythms to undergo systematic investigation (Pittendrigh, 1954; Pittendrigh et al., 1958; Chandrashekaran, 1967; Pittendrigh, 1967; Skopik and Pittendrigh, 1967; Zimmerman et al., 1968) and recent efforts have elucidated its anatomical and physiological basis (Krüger et al., 2015; Selcho et al., 2017) and circadian control of the developmental process (Mark et al., 2021). Further, the act of eclosion is considered a fixed action pattern (Kim et al., 2006), hence compared to other behavioral rhythms such as activity, feeding etc., it is unperturbed by other behavioral outputs, interspecific interactions, or motivational state. However, it is disrupted in core clock mutants such as those of *period* and *timeless* under constant as well as cyclic conditions (Sehgal et al., 1994; Qiu and Hardin, 1996; De et al., 2012; Ruf et al., 2021). As a result, it appears to be a more reliable indicator of core clock output when compared to other rhythms.

The circadian clock of organisms can entrain to various zeitgebers viz. light, temperature, humidity, and social cues. However, most research on circadian rhythms is conducted in the presence of a single cycling zeitgeber under controlled laboratory conditions. In contrast, multiple zeitgebers cycle simultaneously in natural environments (Helm et al., 2017). Although simple laboratory regimes may be appropriate for testing a zeitgeber’s effect under standard conditions, insights from them about organismal rhythms in natural environments are limited. Thus, the action of multiple zeitgebers on entrainment and the phasing of rhythms is an ongoing question in the field.

Studies in the past decade have shown considerable differences in activity-rest and eclosion rhythms under natural conditions from laboratory experiments (De et al., 2012; Vanin et al., 2012; Prabhakaran et al., 2013; Prabhakaran and Sheeba, 2013). Under standard laboratory conditions (LD (12:12), 25°C, 70% RH), distinct morning and evening activity peaks with a siesta during the middle of the day have been observed for *D. melanogaster*. However, under semi-natural conditions, an additional afternoon peak of activity can occur instead of the siesta (Vanin et al., 2012), mainly dependent on temperature, requiring TRPA1 ion channel function (Das et al., 2015; Green et al., 2015). In a study conducted in Europe, PER levels were found to change seasonally under semi-natural conditions, whereas those of TIM remain relatively constant. Thus, the oscillations of PER and TIM proteins become decoupled under warm summer conditions (Menegazzi et al., 2013).

Furthermore, experiments in semi-natural conditions using laboratory-reared, large outbreeding *Drosophila melanogaster* populations have provided ecologically relevant insights. Two sets of long-term laboratory populations with divergent chronotypes termed *Early* and *Late* (Kumar et al., 2007) exhibited greatly enhanced divergence and highly consolidated emergence under semi-natural conditions compared to laboratory conditions (Vaze et al., 2012). The authors speculated it to be a combined effect of multiple zeitgebers and twilight transitions under semi-natural conditions, both of which are absent under standard laboratory conditions. Similarly, laboratory populations selected for accuracy of emergence in a narrow window of time showed an enhanced peak and narrower gate width when assayed under semi-natural conditions compared to LD (12:12) (Kannan et al., 2012). Further, another study compared eclosion rhythms of three closely related *Drosophilids* under semi-natural conditions – *D. melanogaster*, *D. malerkotliana* and *D. ananassae*, known to exhibit differences in the phasing of eclosion under standard laboratory conditions. Surprisingly there was no difference among species in the phasing of eclosion even across different seasons, suggesting that differences in the phase of entrainment of these species are specific to laboratory conditions (Prabhakaran et al., 2013). These results also indicated that there is no certainty that rhythms in complex natural environments will always exhibit higher divergence in the phase-angle of entrainment than in lab conditions.

Recent evidence also indicates the necessity of a functional molecular clock for appropriately timing eclosion under semi-natural conditions (Ruf et al., 2021). According to evolutionary theory, heritable variation is the substrate upon which selection acts and allows for the adaptive evolution of the trait. Thus, the maintenance of genetic variation in clock properties in populations may facilitate adaptation to new selection pressures or environments. With this, we asked if subjecting large outbreeding laboratory populations to rearing under semi-natural conditions could result in changes in the circadian clock i.e. whether adaptation to semi-natural environments altered the circadian phenotype under standard laboratory conditions. If so, what clock properties would evolve to be different? We also hypothesized that specific time cues or aspects of time cues could be more important in terms of the phasing of the eclosion rhythm under semi-natural conditions.

To study how circadian clocks evolve differently in semi-natural conditions compared to standard lab conditions, we reared outbred populations of *Drosophila melanogaster* under semi-natural conditions for 156 generations (NT24) along with control populations (T24) in the laboratory (as of May 2022). Our research location at Jakkur, Bengaluru (13.06° N, 77.62° E), is centrally positioned on the Deccan Plateau in southern India, at a height of 900 m above mean sea level. The climate of Bengaluru is classified as tropical savanna (Peel et al., 2007), with distinct dry and wet seasons. Due to its proximity to the equator, photoperiodic fluctuation is minor compared to temperate conditions, ranging between approx. 11.4 and 12.9 h of daylight throughout the year. Seasonal change occurs as a result of changes in light intensity, rainfall, magnitude of temperature and humidity cycles throughout the year, despite the fact that photoperiodic variation is insignificant (Prabhakaran et al., 2013).

Here, by conducting a series of experiments on the outdoor-reared NT24 populations and laboratory-reared T24 control populations, we show that 1) under laboratory regimes-light-dark cycles and constant conditions, NT24 and T24 populations show similar patterns of phasing and intrinsic free-running period, respectively 2) under semi-natural conditions, NT24 populations exhibit an advanced phase of eclosion compared to T24 controls in a season-dependent manner 3) NT24 populations do not track the timing of a specific environmental variable across all seasons 4) Difference in the phasing of eclosion for NT24 populations compared to T24 appears to be in response to the magnitude of temperature cycle variables.

## Methods

### Fly Populations

**FIGURE 1:**
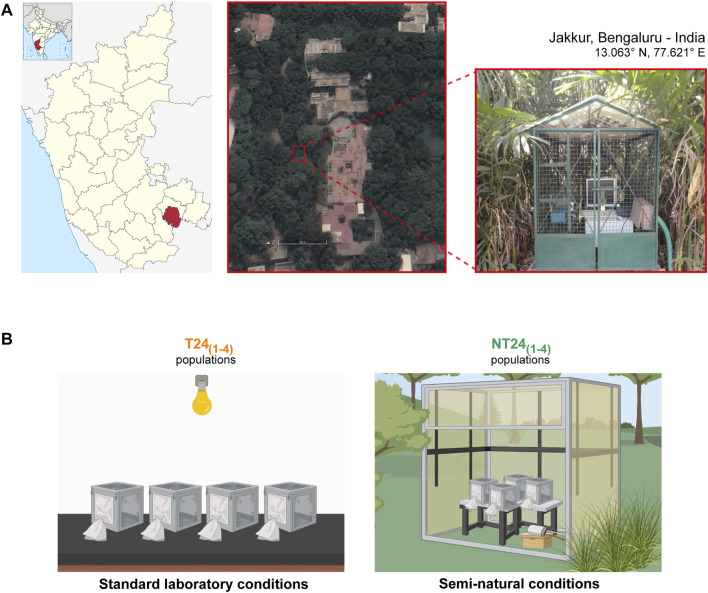
*Drosophila melanogaster* populations used for the study. **(A)** Satellite image (left) and picture (right) of the study site and outdoor enclosure at JNCASR institutional grounds, Jakkur, Bengaluru in India. **(B)** Two sets of *Drosophila melanogaster* populations used: T24 populations (left) were maintained under standard laboratory conditions of LD (12:12), 25°C, ∼70% RH. NT24 populations (right), derived from T24 populations in 2013, were maintained under semi-natural conditions with naturally varying environmental conditions. Adult flies are maintained in Plexiglas cages with food provided *ad libitum* under a 21-days generation cycle (created using BioRender.com).

*Drosophila melanogaster* populations used in this study were initially wild-caught from South Amherst, MA, United States and reared as large outbreeding populations under LL at 25°C for over 700 generations (Joshi and Mueller, 1996; Sheeba et al., 1998), allowing us to have knowledge of the population ancestry and the functional state of circadian clock properties. From these, four large outbreeding *Drosophila melanogaster* populations were derived and maintained in the laboratory for 186 generations under LD (12:12) at 25°C and ∼70% RH called ‘T24 populations’, earlier referred to as LD stocks in (Shindey et al., 2016). The T24_1-4_ populations were used to derive a population each and this new set of populations - NT24_1-4_ was kept under semi-natural conditions in an outdoor enclosure (De et al., 2012), located at the JNCASR campus, Jakkur, Bengaluru, India (13.06° N, 77.62° E; Figure 1). Up to May 2022, the T24 populations have completed 344 generations, while the NT24 populations have been under selection for 156 generations.

A pesticide-free zone was maintained (∼30 m radius), and light intensity, temperature, and relative humidity at the outdoor enclosure were recorded using DEnM (TriKinetics Inc., Waltham, MA, United States). Populations typically consisting of about 1,500 adults (∼1:1 sex ratio) were maintained in plexiglass cages (25 × 20 × 15 cm^3^) on banana-jaggery food medium on a 21-days non-overlapping generation cycle. Before collecting eggs for the next generation, cages were provided with food plates with yeast paste for ∼48 h.

**FIGURE 2:**
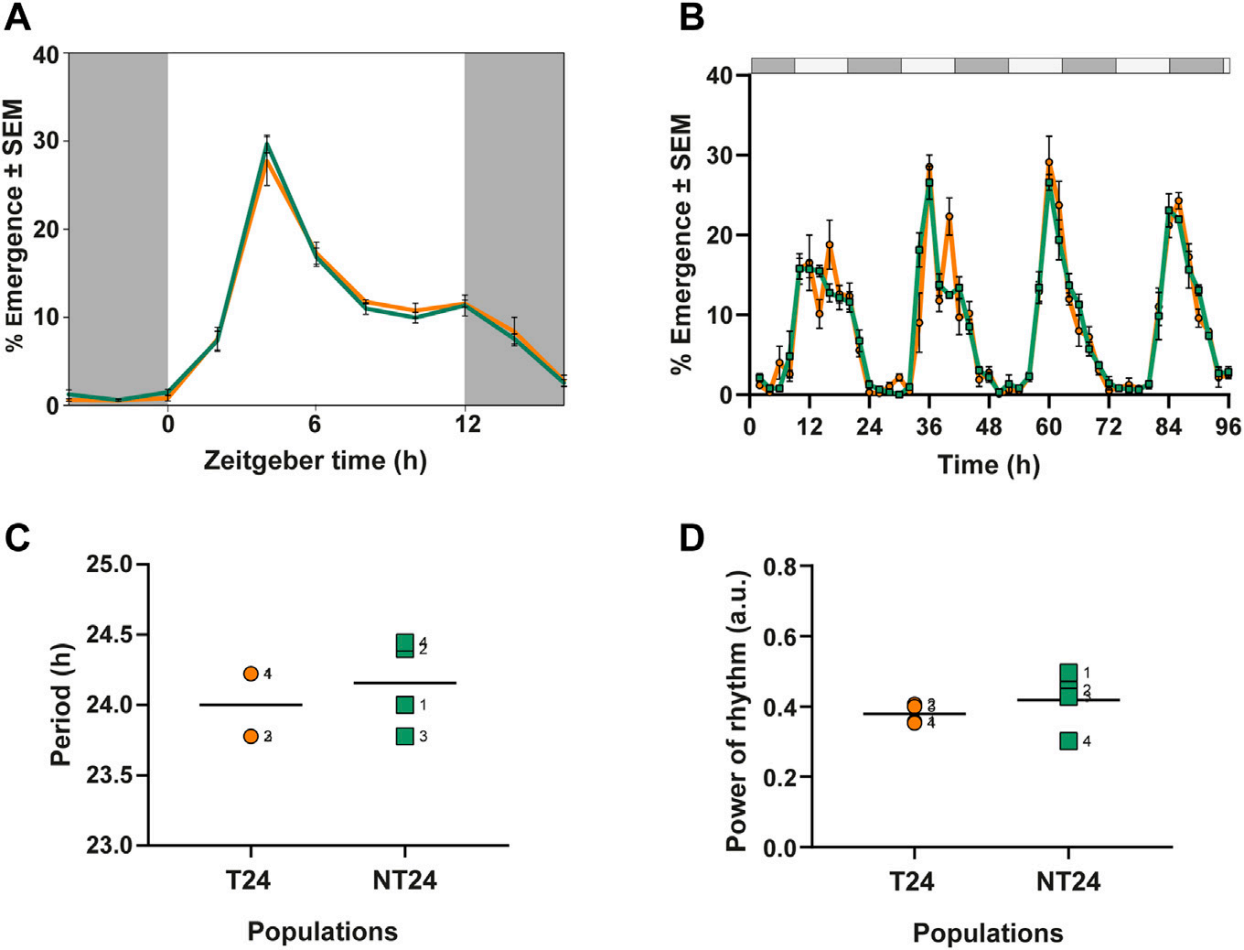
Eclosion rhythm under standard laboratory conditions and constant conditions. **(A)** Eclosion profiles for NT24 (green) and T24 (orange) plotted as daily average percentage of total individuals, averaged across 10 replicate vials under LD (12:12), 25°C, ∼70% RH **(B)** Time series of daily percentage emergence for NT24 and T24 populations under constant darkness (DD), Error bars = SEM **(C)** Free-running period (autocorrelation) under DD **(D)** Power of rhythm (autocorrelation) under DD. For **(C,D)** individual points represent independent replicate populations, dashed line represents mean, Two-Way ANOVA, *p* > 0.05.

**FIGURE 3:**
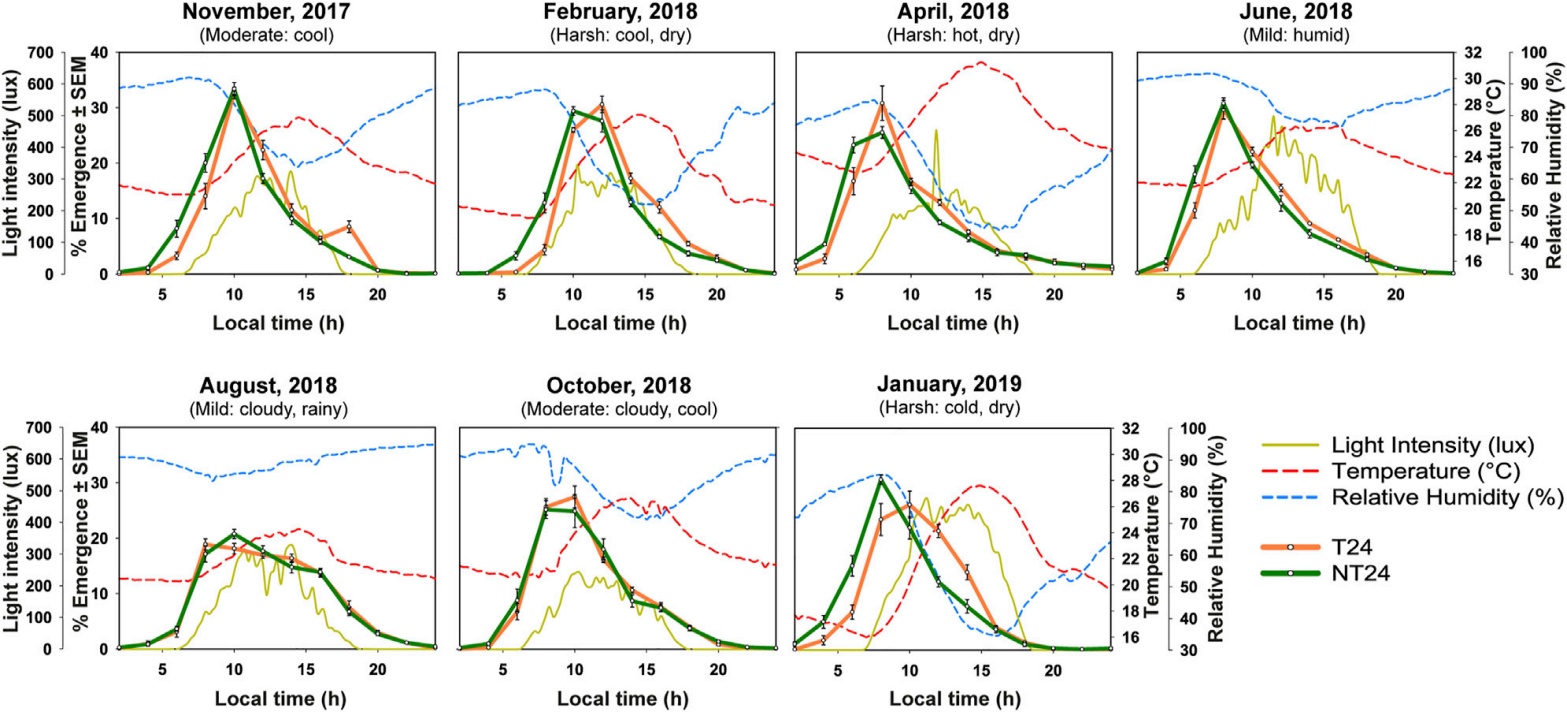
Eclosion rhythm under changing semi-natural conditions (Nov-2017 to Jan-2019). Eclosion profiles for NT24 (green) and T24 (orange) plotted as daily numbers of emerging flies, averaged across 10 replicate vials, for assays conducted across various months of the year with weather description based on existing knowledge of fly preferences. Assays were conducted in 7 different months from 2017 to 2019 under semi-natural conditions and average profiles for environmental variables of light (yellow-solid curve), temperature (red-dashed curve) and humidity (blue-dashed curve).

**FIGURE 4:**
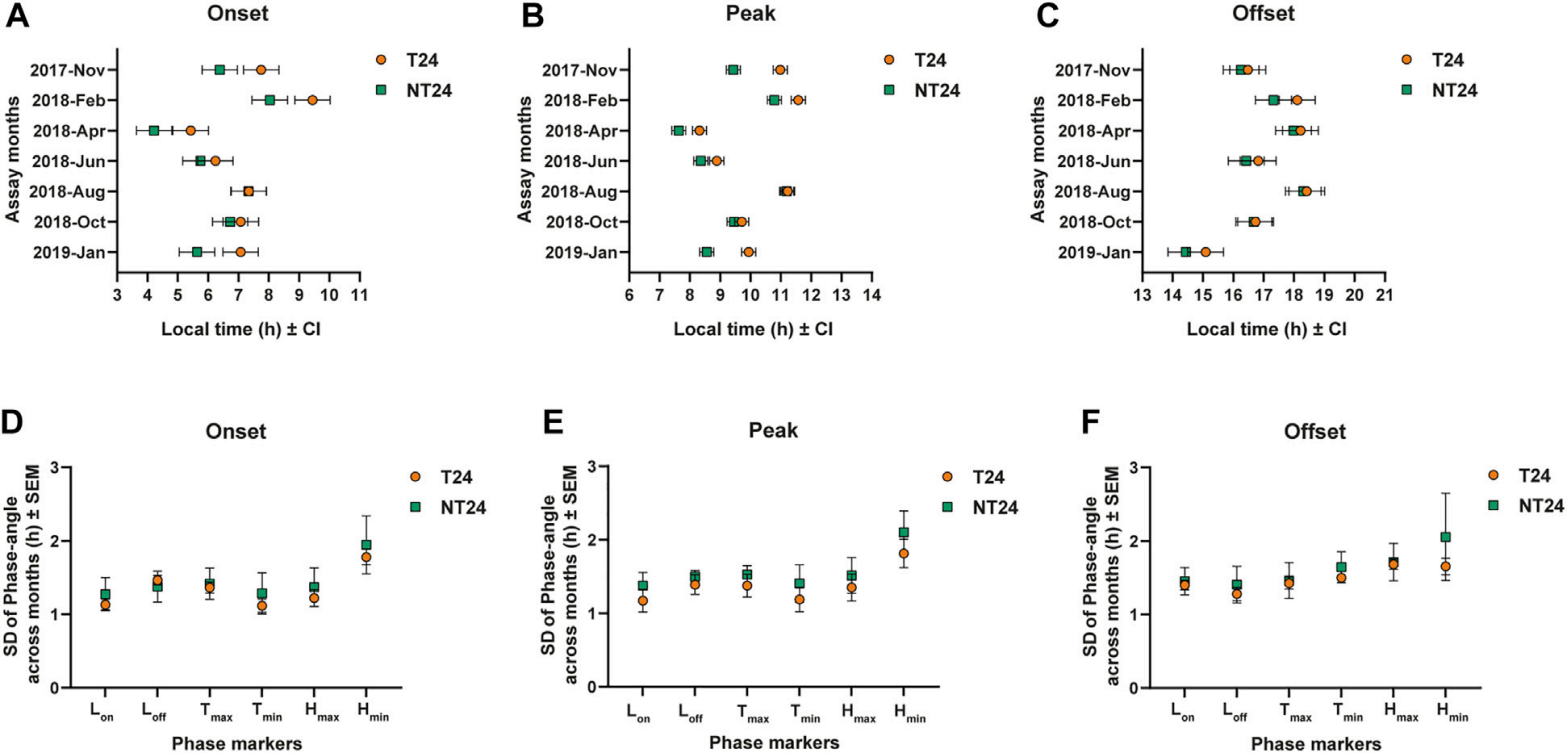
Phasing of the eclosion rhythm under semi-natural conditions and phase-angle variation. **(A)** Average phase of Onset: Three-way ANOVA, *Selection*, *Month*, *Selection* × *Month*; *p* < 0.05, Error bars: 95% CI via Tukey’s HSD (significant for *Selection* × *Month*). **(B)** Average phase of Peak: Three-way ANOVA, *Selection*, *Month*; *p* < 0.05, Error bars: 95% CI via Tukey’s HSD (significant for *Selection*). **(C)** Average phase of Offset: Three-way ANOVA, *Selection*, *Month*; *p* < 0.05, Error bars: 95% CI via Tukey’s HSD (not significant for *Selection*). Variation in phase angle across months for phase markers of environmental variables viz. Lights On (L_ON_), Lights Off (L_OFF_), Maximum temperature (T_MAX_), Minimum temperature (T_MIN_), Maximum humidity (H_MAX_) and Minimum humidity (H_MIN_) for **(D)** average phase of Onset **(E)** average phase of Peak **(F)** average phase of Offset; Two-way ANOVA, *p* > 0.05 for *Selection*.

**FIGURE 5:**
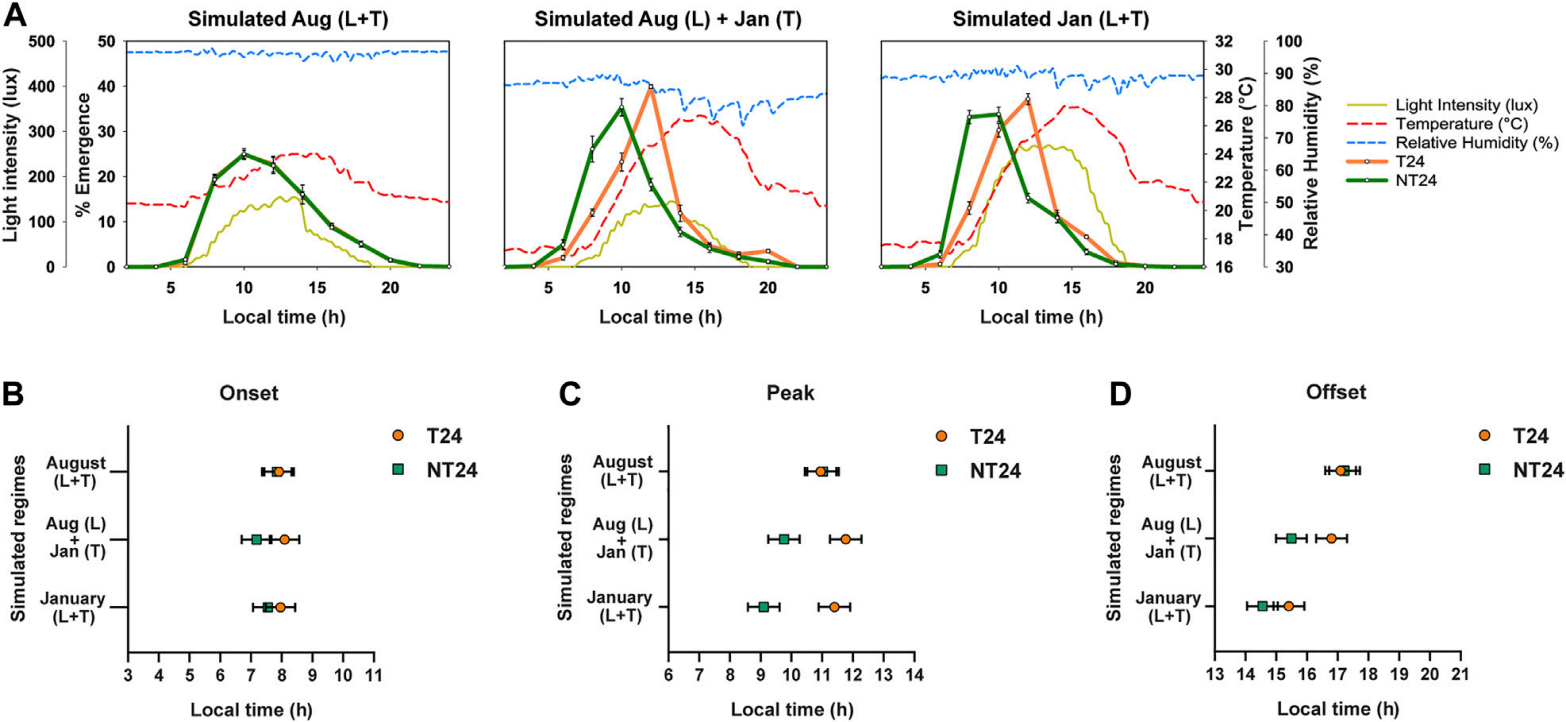
Phasing of the eclosion rhythm under simulated natural light and temperature cycles. **(A)** Eclosion profiles for NT24 (green) and T24 (orange) populations, under three simulated semi-natural conditions in laboratory incubators with average profiles for environmental variables of light (yellow-solid curve), temperature (red-dashed curve) and humidity (blue-dashed curve). Header indicates similarity of simulated regime to that observed in our outdoor enclosure during specific months (as Fig. 2). **(B)** Average phase of Onset: Three-way ANOVA, *Selection*; *p* < 0.05, Error bars: 95% CI via Tukey’s HSD (not significant for *Selection*). **(C)** Average phase of Peak: Three-way ANOVA, *Selection*, *Regime*, *Selection* × *Regime*; *p* < 0.05, Error bars: 95% CI via Tukey’s HSD (significant for *Selection* × *Regime*). **(D)** Average phase of Offset: Three-way ANOVA, *Selection*, *Regime*, *Selection* × *Regime*; *p* < 0.05, Error bars: 95% CI via Tukey’s HSD (significant for *Selection* × *Regime*).

In order to avoid the non-genetic and maternal effects caused due to the rearing regimes on the phenotype being assayed, both sets of populations are maintained together in the ancestral regime (LD (12:12), 25°C, ∼70% RH) for one generation before every experiment. The progeny of these ‘standardized’ populations was used for all the experiments. The experiments described in Figures 2–4 were conducted between 80th—100th generation of NT24_1–4_, whereas those in Figure 5 were conducted between 149th—150th generation. Experiments using either LD (12:12), DD or simulated light and temperature regimes were conducted in environment controlled incubators (DR-36VL, Percival Scientific, Perry, United States).

### Adult Eclosion Rhythm Assay

For the eclosion assay, ∼250 eggs/vial (9 cm height × 2.4 cm diameter) were collected in 10 vials/population, each containing 10 ml of banana-jaggery (BJ) medium, and transferred into the respective assay regime. Upon initiation of emergence, the number of flies emerging every 2 h was recorded for 4 days. This assay was carried out in four different conditions: 1) Standard laboratory conditions- LD (12:12), 25°C, ∼70% RH, 2) Semi-natural conditions (SN)- seasonally varying light, temperature, and humidity, 3) Constant darkness (DD)- 25°C, ∼70% RH 4) Simulated semi-natural conditions with varying light and temperature cycles.

### Quantification of Rhythm Parameters

We quantified 3 phase markers to assess aspects of the rhythm that change under each of the assay settings of entraining regimes (LD/SN): 1) Phase of Onset of eclosion- the time point when the percentage of emerging flies surpassed 5% of total emergence 2) Phase of Offset- the time point when the percentage of emerging flies surpassed 95% of total emergence of the cumulative distribution of eclosion over one cycle/vial 3) Phase of Peak as the time point when the most flies emerged from 1 cycle/vial. If two successive time points had the same maximum number of eclosing flies, the average time between the two time points was calculated for determining the phase of Peak.

### Analysis of Environmental Data

To obtain phase markers for environmental variables under semi-natural conditions, for light, the average and maxima for each day were extracted (since minima of light intensity was 0 lux). For temperature and humidity, average, maxima, minima and amplitude for each day were extracted from environmental recording. Average values of environmental variables across assay days for each month were used to check correlations between environmental variables (Pearson’s correlation coefficient) and for a Principal Component Analysis (PCA). In order to obtain a measure of across-season variation in phase-angle of eclosion (Figures 4D–F), for each assay, we first obtained daily values for each eclosion phase marker (Onset, Peak, Offset) along with daily values of each of the environmental phase markers (L_ON_, L_OFF_, T_MAX_, T_MIN_, H_MAX_, and H_MIN_). Within an assay, the phase-angle averaged across days was calculated for each combination of eclosion and environmental phase marker. From the above data, the standard deviation of average phase-angle values across assays was calculated and statistically compared.

### Data Analysis and Statistics

Values for eclosion rhythm phase markers for LD and SN regimes were computed using custom MATLAB scripts. For analysis of DD data, we estimated the free-running period and power of rhythm using autocorrelation in RhythmicAlly (Abhilash and Sheeba, 2019), based on R (v3.6.3). All data were statistically tested using a randomized block design, mixed model ANOVA approach with *Block* i.e. population as the random factor. Normality of distribution was ascertained using Shapiro-Wilk test. The details of these analyses are mentioned with the results of each assay, and statistical tables are contained in the supplementary file. Tukey’s honest significant difference (HSD) tests were used to perform all post-hoc multiple comparisons. All statistical tests were carried out using STATISTICA v7.0 (StatSoft, Tulsa, OK, United States), as well as the results were deemed significant at *α* = 0.05.

## Results

### Adaptation to Semi-natural Environments has Not Changed Features of Rhythmic Eclosion Under Standard Laboratory Light-Dark Cycles

As an initial step to understand whether rearing under semi-natural conditions had led to phenotypic differences in circadian behavior in their ancestral regime, we assayed the eclosion rhythm of NT24 and T24 populations under standard laboratory conditions (LD). We examined 3 phase markers- Onset, Peak, and Offset of the rhythm and found no difference between NT24 and T24 populations (Figure 2A). Two-way ANOVA with *Selection* and *Block*, phase of Onset: F_1,3_ = 0.643, *p* = 0.481; phase of Peak: F_1,3_ = 0.18, *p* = 0.697; phase of Offset: F_1,3_ = 1.08, *p* = 0.374.

### Selection Under Semi-natural Conditions has Not Changed Free-Running Period and Power of the Eclosion Rhythm Under Constant Conditions

To characterize intrinsic clock properties, we assayed the eclosion rhythm under constant darkness (DD) (Figure 2B). We found that NT24 populations did not differ from T24 in free-running period (Figure 2C) or power of the rhythm (Figure 2D). Two-way ANOVA with *Selection* and *Block*, for free-running period: F_1,3_ = 0.75, *p* = 0.451; power of rhythm: F_1,3_ = 0.998, *p* = 0.391.

### Under Semi-natural Conditions NT24 Populations Have a Season-dependent Advanced Phase of Eclosion due to Earlier Onsets and Peaks

We assayed eclosion rhythm of both sets of populations in our outdoor semi-natural enclosure during various times of the year from November 2017 to January 2019 (Figure 3). As explained in the methods section, we subjected the populations to one generation of common rearing under LD (12:12) (ancestral regime) before all of the assays. We could see that even at a tropical latitude with small changes in photoperiod, various aspects of light, temperature and humidity changed across the assays conducted (see Figure 3). We asked whether these environmental changes may have impacted the eclosion phenotype of NT24 populations since they were reared under semi-natural conditions for at least 80 generations. We find that NT24 populations show an earlier phase of Onset (Figure 4A) and Peak (Figure 4B) in the months of November 2017, February 2018, April 2018, and January 2019 compared to T24 populations, Three-way ANOVA, phase of Onset- *Selection*: F_1,3_ = 136.276, *p* = 0.0014*; Month*: F_6,18_ = 82.701, *p* < 10^–6^; *Selection × Month*: F_6,18_ = 3.813, *p* = 0.013; phase of Peak- *Selection*: F_1,3_ = 818.14, *p* = 9.4 × 10^–5^ *; Month*: F_6,18_ = 58.97, *p* < 10^–6^. Interestingly, there was a strong trend in advance of the phase of Offset across seasons which was not statistically significant between the two sets of populations (Figure 4C), Three-way ANOVA, phase of Offset- *Selection*: F_1,3_ = 27.42, *p* = 0.014, n.s. via Tukey’s HSD*; Month*: F_6,18_ = 21.99, *p* < 10^–6^. The amplitude of temperature cycle was lower in November 2017 (∼5.9°C) compared to other months where we obtained a phase advance. Despite the phasing appearing similar in the average profile (Figure 3) for November 2017, we do find a difference when we calculate phases of Onset and Peak for the NT24 and T24 populations. This is because, despite the phases appearing similar in the average profile (Figure 3), two out of four cycles of eclosion showed a drastic difference in phasing for the NT24 and T24 populations with amplitude of temperature cycle remaining invariant (Supplementary Figure S1).

### The Advancement of Phase of Eclosion of NT24 Populations Is Not due to Tracking of a Specific Environmental Variable Across Seasons

Earlier studies have proposed the role of light intensity and photoperiods in mediating seasonal variation in phase-angle observed across several organisms (Daan and Aschoff, 1975). In our regime, various aspects of light, temperature and humidity change across seasons with comparatively little photoperiodic variation. We wanted to check if NT24 populations track a specific environmental phase marker across seasons and use it to advance the phase of eclosion observed in Figures 3, 4. We hypothesized that this would be reflected in across-season variation in the phase-angle of eclosion such that the variation in NT24 for such an environmental phase marker would be lower than that for T24 populations. We specifically asked if variation in the phase-angle with different environmental phase markers was different between the NT24 and T24 populations. To do this, we compared the standard deviation of phase-angle across months for Onset, Peak, and Offset. We found no significant difference between the NT24 and T24 populations, suggesting that NT24 populations do not track a specific phase marker of light, temperature, and humidity. Three-way ANOVA, for SD of phase of Onset- *Selection*: F_1,3_ = 0.307, *p* = 0.618; *Selection × Phase marker*: F_5,15_ = 1.864, *p* = 0.161; *Phase marker*: F_5,15_ = 129.43, *p* < 10^–6^; for SD of phase of Peak- *Selection*: F_1,3_ = 0.774, *p* = 0.444; *Selection × Phase marker*: F_5,15_ = 0.556, *p* = 0.732; *Phase marker*: F_5,15_ = 158.255, *p* < 10^–6^; for SD of phase of Offset- *Selection*: F_1,3_ = 1.162, *p* = 0.36; *Selection × Phase marker*: F_5,15_ = 1.22, *p* = 0.347; *Phase marker*: F_5,15_ = 4.232, *p* = 0.013.

### NT24 Populations Advance Their Phase of Eclosion Compared to T24 in Response to Increase in Magnitude of Temperature Cycle Variables

Since the intensity of the zeitgeber can alter the phasing of rhythms (Johnson et al., 2003), advance in phases of onset and peak may be altered based on the magnitude of light, temperature and humidity cycles. Analysis of environmental data revealed highly positive or negative correlations among several environmental variables (Supplementary Figure S2A). We then carried out a Principal Component Analysis to better understand the contribution of various environmental variables to the total environmental variation observed across the year (Supplementary Figure S2B). We found that temperature cycle variables (T_max_, T_min_, T_avg_, T_amp_) were major constituents for the primary principal component (Supplementary Table S3). To test the hypothesis that NT24 populations respond differently to temperature cycle shifts in different seasons under semi-natural conditions, we carried out experiments simulating previously observed semi-natural light and temperature regimes in the laboratory. Accordingly, we simulated three regimes (Figure 5A): 1) light and temperature cycles of August 2018; 2) light cycle of August 2018, temperature cycle of January 2019; 3) light and temperature cycles of January 2019. We found a significant difference in phase of onset between the two sets of populations (Figure 5B) via Three-way ANOVA, *Selection*; F_1,3_ = 12.162, *p* = 0.04, however not in the subsequent post-hoc test - Tukey’s HSD (n.s. for *Selection*), *Regime*; F_2,6_ = 1.363, *p* = 0.325, *Selection* × *Regime*; F_2,6_ = 3.137, *p* = 0.117. For phase of Peak, as expected, there was no difference in phasing between the NT24 and T24 populations for the August regime; however, with the change in temperature cycle, NT24 populations significantly advanced their phase (Figure 5C). The January light-temperature regime, however, was not significantly different from the regime with only January temperature cycle, Average phase of Peak: Three-way ANOVA, *Selection*; F_1,3_ = 86.1, *p* = 0.003, *Regime*; F_2,6_ = 10.1, *p* = 0.012, *Selection* × *Regime*; F_2,6_ = 27.9, *p* = 0.0009. We also saw a minor but significant advance in phasing of Offset, although it was not consistent with introduction of January light-temperature regime: Three-way ANOVA, *Selection*; F_1,3_ = 13.59, *p* = 0.035, *Regime*; F_2,6_ = 70.717, *p* = 6.7 × 10^–5^, *Selection* × *Regime*; F_2,6_ = 9.422, *p* = 0.014, Tukey’s HSD (significant for *Selection* × *Regime*). Thus we find that the difference in phasing observed in certain semi-natural regimes can be attributed primarily to the increase in magnitude of the temperature cycle.

## Discussion

While studies on circadian rhythms under naturalistic regimes have been carried out previously, most have not been explicitly designed to provide evolutionary insights. Our study describes an experimental system tailored explicitly for testing such hypotheses (Abhilash and Sharma, 2016). We found that rearing *D. melanogaster* populations under semi-natural conditions resulted in phenotypic change compared to the ancestral controls. Interestingly, we find that adaptation to semi-natural conditions did not alter the circadian phenotype measured under standard laboratory conditions (Figure 2A) and that the two sets of populations did not differ in their intrinsic free-running period or power of the rhythm (Figures 2C,D). This is intriguing as it suggests that a genetic trade-off (Matos et al., 2000) in terms of circadian rhythm phenotypes of phasing and periodicity may not be required for populations adapting to a novel semi-natural environment coming from many generations of laboratory rearing and maintenance.

However, in outdoor experiments, under semi-natural conditions, differences between NT24 and T24 populations were revealed specifically under certain seasons. After conducting experiments in different seasons across the span of 15 months, we found that NT24 populations have a season-dependent advanced phase of eclosion due to advances in the phases of Onset and Peak. These differences were more pronounced under conditions considered harsh for *Drosophila* (Hoffmann, 2010). Though the magnitude of the difference in phasing may appear small (∼1.5 h), we propose that it is biologically significant. This is because 1) we observe consistency of phasing across most replicates (n = 10 vials per population) for each population despite being large and outbred and 2) the advance in phasing occurs at a timing considered ecologically significant for the eclosion rhythm (Pittendrigh, 1954; Cloudsley-Thompson, 1960). Moreover, we also observe an increase in the magnitude of phase difference even under simulated natural light and temperature cycles (∼2 h), consistent with what we saw in natural conditions. One reason for this could be an absence of ultradian fluctuations in simulated conditions in the laboratory compared to the natural environment, which may have enhanced the magnitude of phase advance for NT24 populations, an interesting possibility for future testing.

The motivation to compare variation in phase-angle across seasons was to reveal the relative importance of phasing of an environmental cue compared to the absolute magnitude of the cue for entrainment of an oscillation across seasons. Since there was no difference in across-season variation of phase-angle (Figure 4E), it appears that NT24 populations do not track the timing of a specific environmental variable across seasons for advancing the phase of eclosion. However, the phasing of eclosion rhythm in NT24 flies appears to be sensitive to the magnitude of variables of the temperature cycle. We verified this by checking whether a change in magnitude of the temperature cycle results in the expected differences in phase-angle between the two sets of populations (Figures 5A,B). Indeed, the simulated temperature cycle with greater contrast between T_min_ and T_max_ resulted in an advance in phasing for NT24 populations that remained unchanged with the addition of a similar change in the light cycle (Figure 5D). This suggests that for flies under naturalistic regimes, altered sensory integration as input to the circadian clock may occur due to differences in the magnitude of temperature cycle variables experienced. Previously, similar results have been reported and elucidated for the integration of light input to the clock in various organisms (Lall et al., 2010; Vinayak et al., 2013; Piechura et al., 2017; Woelders et al., 2018). The basis of such altered sensory integration for temperature inputs is currently unknown and requires further investigation.

The differential responsiveness of populations reared under semi-natural conditions to temperature hints that they may be undergoing selection for temperature-directed phasing of eclosion rhythm. Thus, the role of temperature in the presence of other time cues in regulating eclosion rhythm may be more critical than previously thought. Even though it appears that the amplitude of the temperature cycle is a main determinant of advanced phase of eclosion in NT24 populations, other parameters such as rate of change in temperature, temperature cycle range, and so on may also play a role for the same amplitude. This is an interesting premise worth exploring further. While NT24 populations exhibit differential responsiveness to temperature cycles, our experiments till now cannot establish that evolutionarily, temperature cycles in nature alone might have acted as the selection pressure. Thus, while temperature cycles are expected to form an important part of selection on life-history of the NT24 populations, they experience a composite of several cues viz. natural light cycles, humidity cycles etc. along with other micro-environmental changes which may have contributed to the selection pressure experienced over generations.

Apart from light and temperature, humidity has been implicated in influencing the timing of rhythms, however, without much supporting evidence. Eclosion was thought to be mainly limited to the early part of the day as an adaptation to limit water loss and allow optimal wing unfolding (Clayton and Paietta, 1972; Pittendrigh, 1993). Recently, however, humidity cycles (70:30% RH) were insufficient to entrain the eclosion rhythm in *D. melanogaster* lines, and the effect of drastically low humidity (2% RH) on successful eclosion and wing extension was minimal (Ruf et al., 2021). In this context, it seems surprising that NT24 populations, in adapting to semi-natural conditions, have evolved to advance the phase of eclosion to the early morning hours very close to when humidity levels peak (Supplementary Figure S4B). Even though low humidity does not affect successful eclosion and wing extension, one still cannot rule out the possibility of the phase of eclosion determining evolutionary fitness later in life via effects on lifespan or fecundity, and this remains to be tested.

As a natural physical consequence, temperature and humidity cycles are highly correlated and anti-phasic in occurrence (Supplementary Figure S2A). Advancing the phase of eclosion not only makes NT24 populations emerge close to H_max_, but also close to T_min_. Since we find that one of the ways by which NT24 populations advance the eclosion phase is by responding to changes in the magnitude of temperature cues, it would be interesting to test the limits of the range of temperature cycles for which such a response is possible. It is also interesting to speculate if and how the circadian clock could differentiate sudden and potentially harmful changes in temperature from the necessary temperature changes required for phasing eclosion rhythm output. Although there have been recent advances in the characterization of peripheral clocks in temperature entrainment and elucidation of the differential role of ion channels at different temperatures, our knowledge of temperature entrainment in *Drosophila* is still limited (George and Stanewsky, 2021).

By virtue of their climate, tropical regions harbor more arthropod species biodiversity and number (Stork, 2018). Thus, it is equally important to study insect behavior and rhythms under tropical naturalistic habitats with lower photoperiodic variation and different temperature ranges. Our study reports the results of long-term experimental evolution in such conditions and the intricacies associated with the effect of timing and magnitude of environmental variables on the phasing of the eclosion rhythm. An alternative approach to ours could be introducing wild-caught populations to laboratory and captive-natural environments simultaneously and assessing adaptation to both in parallel. Overall, in order to derive any general principles that may operate in real-world conditions, it will be important for such studies to be conducted under a large number of distinct ecological conditions. Future work in this direction will hopefully unravel the mechanistic basis of the evolutionary differences we observe and help us gain a more holistic perspective.

## Data Availability

The raw data supporting the conclusions of this article will be made available by the authors, without undue reservation.
