# Supplementary material for: Drosophila Populations Reared Under Tropical Semi-natural Conditions Evolve Season-dependent Differences in Timing of Eclosion
Source: Front Physiol. 2022 Jul 15;13:954731. doi: 10.3389/fphys.2022.954731 (PMC9334559; doi:10.3389/fphys.2022.954731)
Supplement: Supplementary file 1 [file DataSheet1.docx]

***Drosophila* Populations Reared Under Tropical Semi-natural Conditions Evolve Season-dependent Differences in Timing of Eclosion**

Chitrang Dani and Vasu Sheeba^*^

**Affiliation**: Chronobiology and Behavioural Neurogenetics Laboratory, Neuroscience Unit, Jawaharlal Nehru Centre for Advanced Scientific Research, Bengaluru, India.

***Corresponding author**: Sheeba Vasu

**Email:** [sheeba@jncasr.ac.in](mailto:sheeba@jncasr.ac.in)

**Figure S1**





**Fig.S1** Time-series (raw data) of eclosion, averaged across blocks, in November 2017 under semi-natural conditions corresponding to the figure subset in Fig.3. Y-axis indicates the number of eclosing flies, X-axis indicates the local time in hours. Recorded temperature (dotted line) across days has been plotted along with the time-series for NT24 and T24 populations.

**Figure S2**


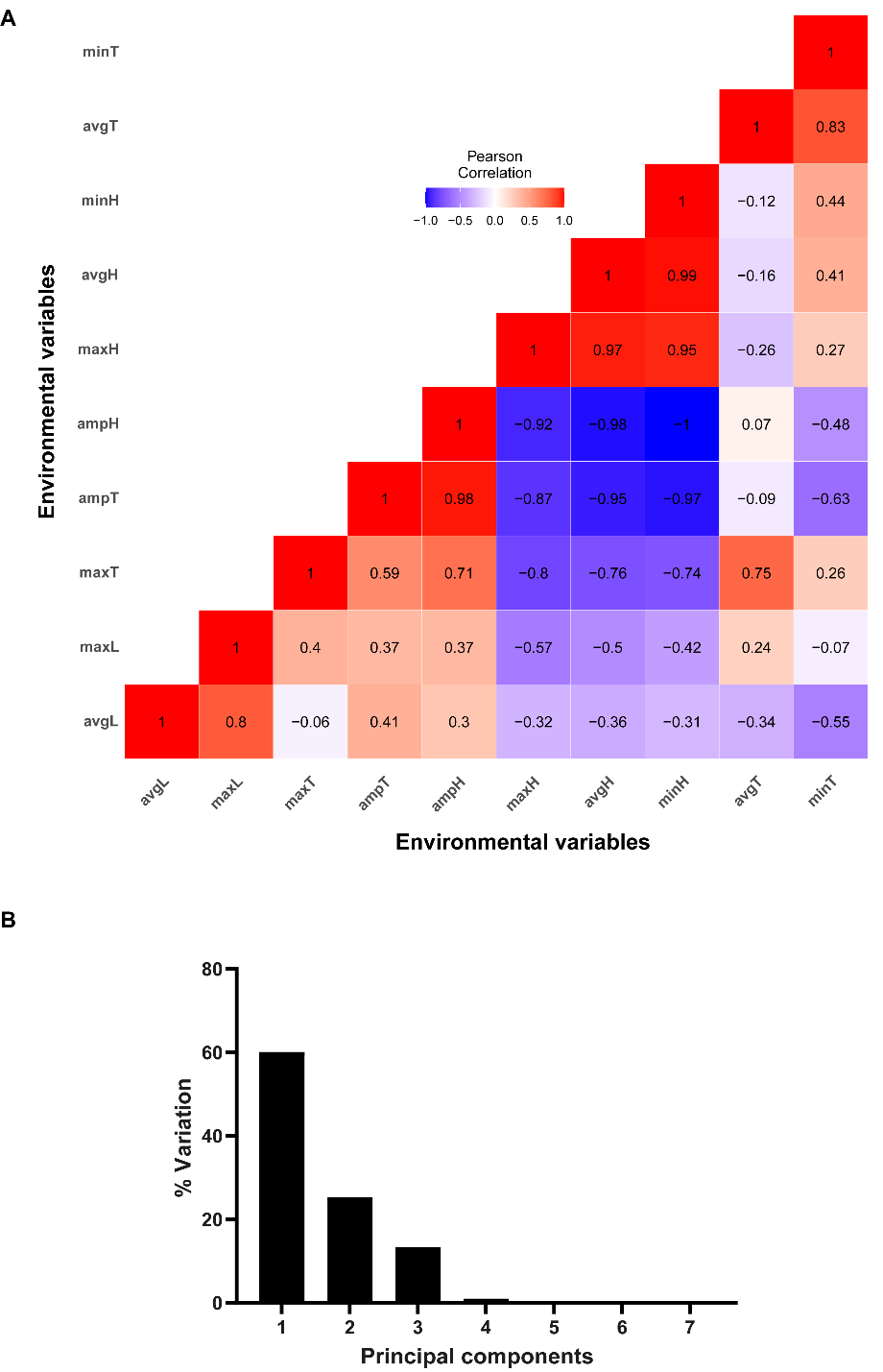


**Fig. S2** A) Heatmap correlation matrix of environmental variables using Pearson’s correlation coefficient B) Histogram showing percentage of variation explained by various principal components derived from environmental variables: PC1 (60.06%), PC2 (25.3%), PC3 (13.4%), PC4 (1%), PC5 (0.2%), PC6 and PC7 (0%).

**Table S3**

|  | **PC1** | **PC2** | **PC3** | **PC4** | **PC5** | **PC6** | **PC7** |
| --- | --- | --- | --- | --- | --- | --- | --- |
| L_avg_ | -0.25335 | 0.16699 | 0.259808 | 0.097753 | -0.00067 | -0.00179 | 0.0000559 |
| L_max_ | -7.13336 | 0.171101 | -0.03733 | -0.04071 | 0.0000732 | 0.0005 | -0.0000116 |
| T_avg_ | 1.412908 | 0.117591 | -0.02804 | -0.05857 | -0.00029 | -0.00652 | 0.000865 |
| T_amp_ | 1.826569 | 0.244255 | 0.010026 | -0.05341 | 0.004201 | 0.009802 | 0.00082 |
| T_max_ | 1.311052 | 0.11417 | -0.04151 | -0.03088 | 0.002916 | -0.00298 | 0.00229 |
| T_min_ | 1.482119 | 0.114616 | -0.01968 | -0.07859 | -0.00573 | -0.0048 | -0.00197 |
| H_avg_ | -0.00699 | -0.42088 | -0.01762 | 0.027096 | -0.01354 | 0.003492 | 0.00114 |
| H_amp_ | 1.272233 | 0.302663 | -0.11748 | 0.118378 | -0.0015 | 0.002867 | -0.00171 |
| H_max_ | -0.31829 | -0.37627 | -0.07876 | 0.119222 | 0.008745 | -0.00284 | 0.000124 |
| H_min_ | 0.407113 | -0.43423 | 0.070579 | -0.10028 | 0.005804 | 0.002275 | -0.00161 |

**Table S3** Loading values for environmental variables (vertical) in the Principal Component Analysis. Various principal components (horizontal) are represented as PC1 for component 1, PC2 for component 2 etc.

**Figure S4**


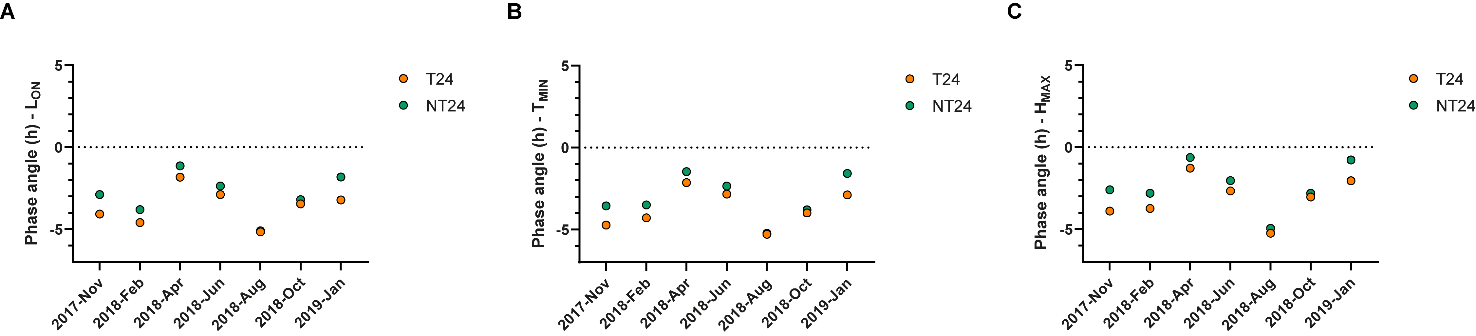


**Fig.S4** Phase-angle for assay months with Peak of eclosion and environmental phase markers of early daytime A) Lights-On (L_ON_) B) Temperature-Minima (T_MIN_) C) Humidity-Maxima (H_MAX_)

List of ANOVA tables

1

(associated data for Fig. 2A)

| Univariate Tests of Significance for ONSET - Over-parameterized model Type III decomposition | | | | | | | | |
| --- | --- | --- | --- | --- | --- | --- | --- | --- |
|  | Effect | SS | Df | MS | Den.Syn. error df | Den.Syn. | F | p |
| Intercept | Fixed | 756.569 | 1 | 756.569 | 3 | 1.073584 | 704.7133 | 0.000117 |
| SEL | Fixed | 0.2059 | 1 | 0.2059 | 3 | 0.320292 | 0.6428 | 0.481342 |
| BLOCK | Random | 3.2208 | 3 | 1.0736 | 3 | 0.320292 | 3.3519 | 0.173522 |
| SEL*BLOCK | Random | 0.9609 | 3 | 0.3203 | 0 | 0 |  |  |
| Error |  |  | 0 |  |  |  |  |  |

| Univariate Tests of Significance for PEAK - Over-parameterized model Type III decomposition | | | | | | | | |
| --- | --- | --- | --- | --- | --- | --- | --- | --- |
|  | Effect | SS | Df | MS | Den.Syn. error df | Den.Syn. | F | p |
| Intercept | Fixed | 1367.572 | 1 | 1367.572 | 3 | 0.034713 | 39396.66 | <10^-6^ |
| SEL | Fixed | 0.06 | 1 | 0.06 | 3 | 0.326061 | 0.18 | 0.697351 |
| BLOCK | Random | 0.104 | 3 | 0.035 | 3 | 0.326061 | 0.11 | 0.950821 |
| SEL*BLOCK | Random | 0.978 | 3 | 0.326 | 0 | 0 |  |  |
| Error |  |  | 0 |  |  |  |  |  |

| Univariate Tests of Significance for OFFSET - Over-parameterized model Type III decomposition | | | | | | | | |
| --- | --- | --- | --- | --- | --- | --- | --- | --- |
|  | Effect | SS | Df | MS | Den.Syn. error df. | Den.Syn. | F | p |
| Intercept | Fixed | 3772.185 | 1 | 3772.185 | 3 | 0.146809 | 25694.54 | 0.000001 |
| SEL | Fixed | 0.009 | 1 | 0.009 | 3 | 0.00832 | 1.08 | 0.374474 |
| BLOCK | Random | 0.44 | 3 | 0.147 | 3 | 0.00832 | 17.65 | 0.020744 |
| SEL*BLOCK | Random | 0.025 | 3 | 0.008 | 0 | 0 |  |  |
| Error |  |  | 0 |  |  |  |  |  |

**2**

(associated data for Fig. 2C)

| Univariate Tests of Significance for Period - Over-parameterized model Type III decomposition | | | | | | | | |
| --- | --- | --- | --- | --- | --- | --- | --- | --- |
|  | Effect | SS | Df | MS | Den.Syn. error df | Den.Syn. | F | p |
| Intercept | Fixed | 4637.915 | 1 | 4637.915 | 3.000000 | 0.104362 | 44440.59 | <10^-6^ |
| SEL | Fixed | 0.048 | 1 | 0.048 | 3.000000 | 0.064856 | 0.75 | 0.451221 |
| BLOCK | Random | 0.313 | 3 | 0.104 | 3.000000 | 0.064856 | 1.61 | 0.352734 |
| SEL*BLOCK | Random | 0.195 | 3 | 0.065 | 0.000000 | 0.000000 |  |  |
| Error |  |  | 0 |  |  |  |  |  |

3

(associated data for Fig. 2D)

| Univariate Tests of Significance for Power - Over-parameterized model Type III decomposition | | | | | | | | |
| --- | --- | --- | --- | --- | --- | --- | --- | --- |
|  | Effect | SS | Df | MS | Den.Syn. error df | Den.Syn. | F | p |
| Intercept | Fixed | 1.270749 | 1 | 1.270749 | 3.000000 | 0.004571 | 277.9745 | 0.000470 |
| SEL | Fixed | 0.003037 | 1 | 0.003037 | 3.000000 | 0.003042 | 0.9982 | 0.391373 |
| BLOCK | Random | 0.013714 | 3 | 0.004571 | 3.000000 | 0.003042 | 1.5026 | 0.373020 |
| SEL*BLOCK | Random | 0.009127 | 3 | 0.003042 | 0.000000 | 0.000000 |  |  |
| Error |  |  | 0 |  |  |  |  |  |

4

(associated data for Fig. 4A)

| Univariate Tests of Significance for ONSET - Over-parameterized model Type III decomposition | | | | | | | | |
| --- | --- | --- | --- | --- | --- | --- | --- | --- |
|  | Effect | SS | Df | MS | Den.Syn. error df | Den.Syn. | F | p |
| Intercept | Fixed | 2546.627 | 1 | 2546.627 | 3 | 0.762781 | 3338.61 | 0.000011 |
| **SEL** | **Fixed** | **11.265** | **1** | **11.265** | **3** | **0.082664** | **136.276** | **0.00135** |
| BLOCK | Random | 2.288 | 3 | 0.763 | 0.31581 | 0.040943 | 18.63 | 0.482092 |
| **MONTH** | **Fixed** | **70.939** | **6** | **11.823** | **18** | **0.142961** | **82.701** | **<10^-6^** |
| SEL*BLOCK | Random | 0.248 | 3 | 0.083 | 18 | 0.184682 | 0.448 | 0.722015 |
| **SEL*MONTH** | **Fixed** | **4.225** | **6** | **0.704** | **18** | **0.184682** | **3.813** | **0.012534** |
| BLOCK*MONTH | Random | 2.573 | 18 | 0.143 | 18 | 0.184682 | 0.774 | 0.70367 |
| SEL*BLOCK*MONTH | Random | 3.324 | 18 | 0.185 | 0 | 0 |  |  |
| Error |  |  | 0 |  |  |  |  |  |

**5**

(associated data for Fig. 4B)

| Univariate Tests of Significance for PEAK - Over-parameterized model Type III decomposition | | | | | | | | |
| --- | --- | --- | --- | --- | --- | --- | --- | --- |
|  | Effect | SS | Df | MS | Den.Syn. error df | Den.Syn. | F | p |
| Intercept | Fixed | 5287.972 | 1 | 5287.972 | 3 | 0.05648 | 93624.84 | <10^-6^ |
| **SEL** | **Fixed** | **7.786** | **1** | **7.786** | **3** | **0.009516** | **818.14** | **0.000094** |
| BLOCK | Random | 0.169 | 3 | 0.056 | 0 | - | - | - |
| **MONTH** | **Fixed** | **72.577** | **6** | **12.096** | **18** | **0.205138** | **58.97** | **<10^-6^** |
| SEL*BLOCK | Random | 0.029 | 3 | 0.01 | 18 | 0.310943 | 0.03 | 0.99254 |
| SEL*MONTH | Fixed | 3.707 | 6 | 0.618 | 18 | 0.310943 | 1.99 | 0.121042 |
| BLOCK*MONTH | Random | 3.692 | 18 | 0.205 | 18 | 0.310943 | 0.66 | 0.807055 |
| SEL*BLOCK*MONTH | Random | 5.597 | 18 | 0.311 | 0 | 0 |  |  |
| Error |  |  | 0 |  |  |  |  |  |

**6**

(associated data for Fig. 4C)

| Univariate Tests of Significance for OFFSET - Over-parameterized model Type III decomposition | | | | | | | | |
| --- | --- | --- | --- | --- | --- | --- | --- | --- |
|  | Effect | SS | Df | MS | Den.Syn. error df | Den.Syn. | F | p |
| Intercept | Fixed | 16085.36 | 1 | 16085.36 | 3 | 0.309098 | 52039.66 | <10^-6^ |
| **SEL** | **Fixed** | **1.72** | **1** | **1.72** | **3** | **0.062764** | **27.42** | **0.013557** |
| BLOCK | Random | 0.93 | 3 | 0.31 | 12.59546 | 0.495563 | 0.62 | 0.612576 |
| **MONTH** | **Fixed** | **73.64** | **6** | **12.27** | **18** | **0.558207** | **21.99** | **<10^-6^** |
| SEL*BLOCK | Random | 0.19 | 3 | 0.06 | 18 | 0.125408 | 0.5 | 0.686667 |
| SEL*MONTH | Fixed | 0.9 | 6 | 0.15 | 18 | 0.125408 | 1.2 | 0.35185 |
| **BLOCK*MONTH** | **Random** | **10.05** | **18** | **0.56** | **18** | **0.125408** | **4.45** | **0.001366** |
| SEL*BLOCK*MONTH | Random | 2.26 | 18 | 0.13 | 0 | 0 |  |  |
| Error |  |  | 0 |  |  |  |  |  |

7

(associated data for Fig. 4D)

| Univariate Tests of Significance for SD ONSET - Over-parameterized model Type III decomposition | | | | | | | | |
| --- | --- | --- | --- | --- | --- | --- | --- | --- |
|  | Effect | SS | Df | MS | Den.Syn. error df | Den.Syn. | F | p |
| Intercept | Fixed | 93.44122 | 1 | 93.44122 | 3 | 0.184392 | 506.7536 | 0.000192 |
| SEL | Fixed | 0.11538 | 1 | 0.11538 | 3 | 0.375655 | 0.3071 | 0.618075 |
| BLOCK | Random | 0.55318 | 3 | 0.18439 | 2.88515 | 0.368429 | 0.5005 | 0.708886 |
| **PHASE MARKER** | **Fixed** | **2.42861** | **5** | **0.48572** | **15** | **0.003753** | **129.431** | **<10^-6^** |
| SEL*BLOCK | Random | 1.12697 | 3 | 0.37566 | 15 | 0.010978 | 34.2174 | 0.000001 |
| SEL*PHASE MARKER | Fixed | 0.10232 | 5 | 0.02046 | 15 | 0.010978 | 1.864 | 0.160769 |
| BLOCK*PHASE MARKER | Random | 0.05629 | 15 | 0.00375 | 15 | 0.010978 | 0.3418 | 0.977198 |
| SEL*BLOCK*PHASE MARKER | Random | 0.16468 | 15 | 0.01098 | 0 | 0 |  |  |
| Error |  |  | 0 |  |  |  |  |  |

**8**

(associated data for Fig. 4E)

| Univariate Tests of Significance for SD PEAK - Over-parameterized model Type III decomposition | | | | | | | | |
| --- | --- | --- | --- | --- | --- | --- | --- | --- |
|  | Effect | SS | Df | MS | Den.Syn. error df | Den.Syn. | F | p |
| Intercept | Fixed | 104.5884 | 1 | 104.5884 | 3.00000 | 0.284457 | 367.6775 | 0.000310 |
| SEL | Fixed | 0.4277 | 1 | 0.4277 | 3.00000 | 0.552325 | 0.7744 | 0.443656 |
| BLOCK | Random | 0.8534 | 3 | 0.2845 | 2.88472 | 0.541645 | 0.5252 | 0.696001 |
| **PHASE MARKER** | **Fixed** | **2.4680** | **5** | **0.4936** | **15.00000** | **0.003119** | **158.2546** | **<10^-6^** |
| SEL*BLOCK | Random | 1.6570 | 3 | 0.5523 | 15.00000 | 0.013799 | 40.0264 | <10^-6^ |
| SEL*PHASE MARKER | Fixed | 0.0384 | 5 | 0.0077 | 15.00000 | 0.013799 | 0.5563 | 0.731687 |
| BLOCK*PHASE MARKER | Random | 0.0468 | 15 | 0.0031 | 15.00000 | 0.013799 | 0.2260 | 0.996714 |
| SEL*BLOCK*PHASE MARKER | Random | 0.2070 | 15 | 0.0138 | 0.00000 | 0.000000 |  |  |
| Error |  |  | 0 |  |  |  |  |  |

**9**

(associated data for Fig. 4F)

| Univariate Tests of Significance for SD OFFSET - Over-parameterized model Type III decomposition | | | | | | | | |
| --- | --- | --- | --- | --- | --- | --- | --- | --- |
|  | Effect | SS | Df | MS | Den.Syn. error df | Den.Syn. | F | p |
| Intercept | Fixed | 116.4518 | 1 | 116.4518 | 3 | 0.081005 | 1437.595 | 0.00004 |
| SEL | Fixed | 0.2096 | 1 | 0.2096 | 3 | 0.180277 | 1.162 | 0.359924 |
| BLOCK | Random | 0.243 | 3 | 0.081 | 4.21675 | 0.217576 | 0.372 | 0.778105 |
| **PHASE MARKER** | **Fixed** | **1.474** | **5** | **0.2948** | **15** | **0.069651** | **4.232** | **0.013383** |
| SEL*BLOCK | Random | 0.5408 | 3 | 0.1803 | 15 | 0.032352 | 5.572 | 0.008997 |
| SEL*PHASE MARKER | Fixed | 0.1973 | 5 | 0.0395 | 15 | 0.032352 | 1.22 | 0.347377 |
| BLOCK*PHASE MARKER | Random | 1.0448 | 15 | 0.0697 | 15 | 0.032352 | 2.153 | 0.074456 |
| SEL*BLOCK*PHASE MARKER | Random | 0.4853 | 15 | 0.0324 | 0 | 0 |  |  |
| Error |  |  | 0 |  |  |  |  |  |

**10**

(associated data for Fig. 5A)

| Univariate Tests of Significance for ONSET - Over-parameterized model Type III decomposition | | | | | | | | |
| --- | --- | --- | --- | --- | --- | --- | --- | --- |
|  | Effect | SS | Df | MS | Den.Syn. error df | Den.Syn. | F | p |
| Intercept | Fixed | 1444.214 | 1 | 1444.214 | 3 | 0.221341 | 6524.83 | 0.000004 |
| SEL | Fixed | 1.286 | 1 | 1.286 | 3 | 0.105773 | 12.162 | 0.03984 |
| BLOCK | Random | 0.664 | 3 | 0.221 | 0.838948 | 0.077154 | 2.869 | 0.440646 |
| REGIME | Fixed | 0.232 | 2 | 0.116 | 6 | 0.085158 | 1.363 | 0.325062 |
| SEL*BLOCK | Random | 0.317 | 3 | 0.106 | 6 | 0.113777 | 0.93 | 0.481998 |
| SEL*REGIME | Fixed | 0.714 | 2 | 0.357 | 6 | 0.113777 | 3.137 | 0.116803 |
| BLOCK*REGIME | Random | 0.511 | 6 | 0.085 | 6 | 0.113777 | 0.748 | 0.63302 |
| SEL*BLOCK*REGIME | Random | 0.683 | 6 | 0.114 | 0 | 0 |  |  |
| Error |  |  | 0 |  |  |  |  |  |

**11**

(associated data for Fig. 5B)

| Univariate Tests of Significance for PEAK - Over-parameterized model Type III decomposition | | | | | | | | |
| --- | --- | --- | --- | --- | --- | --- | --- | --- |
|  | Effect | SS | Df | MS | Den.Syn. error df | Den.Syn. | F | p |
| Intercept | Fixed | 2731.205 | 1 | 2731.205 | 3 | 0.020542 | 132957.1 | <10^-6^ |
| SEL | Fixed | 11.952 | 1 | 11.952 | 3 | 0.138825 | 86.1 | 0.002649 |
| BLOCK | Random | 0.062 | 3 | 0.021 | 1.574244 | 0.131563 | 0.2 | 0.916232 |
| REGIME | Fixed | 2.295 | 2 | 1.148 | 6 | 0.113415 | 10.1 | 0.011957 |
| SEL*BLOCK | Random | 0.416 | 3 | 0.139 | 6 | 0.120676 | 1.2 | 0.40247 |
| SEL*REGIME | Fixed | 6.737 | 2 | 3.369 | 6 | 0.120676 | 27.9 | 0.000914 |
| BLOCK*REGIME | Random | 0.68 | 6 | 0.113 | 6 | 0.120676 | 0.9 | 0.529063 |
| SEL*BLOCK*REGIME | Random | 0.724 | 6 | 0.121 | 0 | 0 |  |  |
| Error |  |  | 0 |  |  |  |  |  |

**12**

(associated data for Fig. 5C)

| Univariate Tests of Significance for OFFSET - Over-parameterized model Type III decomposition | | | | | | | | |
| --- | --- | --- | --- | --- | --- | --- | --- | --- |
|  | Effect | SS | Df | MS | Den.Syn. error df | Den.Syn. | F | p |
| Intercept | Fixed | 6216.36 | 1 | 6216.36 | 3 | 0.75933 | 8186.637 | 0.000003 |
| SEL | Fixed | 2.766 | 1 | 2.766 | 3 | 0.203513 | 13.59 | 0.034602 |
| BLOCK | Random | 2.278 | 3 | 0.759 | 2.632634 | 0.223636 | 3.395 | 0.190334 |
| REGIME | Fixed | 19.016 | 2 | 9.508 | 6 | 0.134452 | 70.717 | 0.000067 |
| SEL*BLOCK | Random | 0.611 | 3 | 0.204 | 6 | 0.11433 | 1.78 | 0.250771 |
| SEL*REGIME | Fixed | 2.154 | 2 | 1.077 | 6 | 0.11433 | 9.422 | 0.014088 |
| BLOCK*REGIME | Random | 0.807 | 6 | 0.134 | 6 | 0.11433 | 1.176 | 0.424501 |
| SEL*BLOCK*REGIME | Random | 0.686 | 6 | 0.114 | 0 | 0 |  |  |
| Error |  |  | 0 |  |  |  |  |  |
